# Ribosomes and Ribosomal Proteins Promote Plasticity and Stemness Induction in Glioma Cells via Reprogramming

**DOI:** 10.3390/cells11142142

**Published:** 2022-07-07

**Authors:** Takuichiro Hide, Ichiyo Shibahara, Madoka Inukai, Ryota Shigeeda, Toshihiro Kumabe

**Affiliations:** Department of Neurosurgery, Kitasato University School of Medicine, 1-15-1 Kitasato, Minami-ku, Sagamihara 252-0374, Japan; ichiyooo@hotmail.com (I.S.); madoca@mtg.biglobe.ne.jp (M.I.); geshi.rugby.no2@gmail.com (R.S.); kuma@kitasato-u.ac.jp (T.K.)

**Keywords:** ribosomal protein, reprogramming, glioblastoma, extra-ribosomal function, microenvironment, glioma stem cell, ribosomal protein S6, ribosome biogenesis, transdifferentiation, plasticity

## Abstract

Glioblastoma multiforme (GBM) is a lethal tumor that develops in the adult brain. Despite advances in therapeutic strategies related to surgical resection and chemo-radiotherapy, the overall survival of patients with GBM remains unsatisfactory. Genetic research on mutation, amplification, and deletion in GBM cells is important for understanding the biological aggressiveness, diagnosis, and prognosis of GBM. However, the efficacy of drugs targeting the genetic abnormalities in GBM cells is limited. Investigating special microenvironments that induce chemo-radioresistance in GBM cells is critical to improving the survival and quality of life of patients with GBM. GBM cells acquire and maintain stem-cell-like characteristics via their intrinsic potential and extrinsic factors from their special microenvironments. The acquisition of stem-cell-like phenotypes and aggressiveness may be referred to as a reprogramming of GBM cells. In addition to protein synthesis, deregulation of ribosome biogenesis is linked to several diseases including cancer. Ribosomal proteins possess both tumor-promotive and -suppressive functions as extra-ribosomal functions. Incorporation of ribosomes and overexpression of ribosomal protein S6 reprogram and induce stem-cell-like phenotypes in GBM cells. Herein, we review recent literature and our published data on the acquisition of aggressiveness by GBM and discuss therapeutic options through reprogramming.

## 1. Introduction

The chemo-radioresistant characteristics and high recurrence potential of glioblastoma multiforme (GBM) pose major clinical problems in its treatment. Thus, the mean survival time of patients with GBM is approximately 1 year, and the 5-year overall survival rate is only 9.8% [1,2]. To improve the prognosis of patients with GBM, innovation in both the surgical tumor resection and chemo-radiotherapy is critical. GBM was classified into GBM, IDH-wildtype (primary GBM) and GBM, IDH-mutant (secondary GBM) in the fourth edition of the World Health Organization (WHO) classification of tumors of the central nervous system published in 2016 [3]. In the fifth edition published in 2021, GMB was only mentioned as GBM, IDH-wildtype; conversely, GBM, IDH-mutant was classified into astrocytoma, IDH-mutant [4].

GBM contains a small population of characteristic cells possessing stem-cell-like features, such as self-renewal, multilineage differentiation, and tumor initiation [5]. This population of cells, referred to as glioma stem cells (GSCs), is considered to be the cause of resistance to chemo-radiotherapy and the consequent recurrence. Investigations on the GSCs derived from GBM tissue, several types of induced GSC-like cells, genetically engineered mouse GBM models, and GBM organoid models have been performed to elucidate the mechanisms of the development, progression, therapeutic resistance, and recurrence of GBM [6,7,8,9,10]. 

A comprehensive analysis using microarrays revealed intratumoral, intertumoral, and spatiotemporal heterogeneities in GBM [11,12,13,14,15]. Moreover, a single-cell RNA-sequencing analysis provided deep insights into the heterogeneity of GBM cells through dynamic alterations in the gene expression patterns [16,17]. Thus, the diversity and plasticity of GBM cells are the causes of therapeutic resistance and recurrence, which are fundamental issues that need to be elucidated [11,18].

In the dynamic phenotypical alteration of glioma cells, the tumor microenvironment through secreted molecules (growth factors, cytokines, chemokines, and extracellular vesicles [EVs]), cell–cell adhesion, cell–extracellular matrix contact, and exposure to physiological conditions (hypoxia, hypothermia, and starvation) plays important roles [19,20,21,22,23,24,25]. To acquire aggressiveness, GBM cells also exploit stimuli from other GBM cells and non-GBM cells such as neurons, astrocytes, oligodendrocytes, macrophages or microglia, immune cells, and endothelial cells [20,21,26,27,28]. Thus, various kinds of stimuli in the tumor microenvironment of GBM can modulate the phenotypes of GBM cells. A clinical challenge in GBM is the high plasticity or reprogramming potential of GBM cells.

Interestingly, Ito et al. reported that incorporating ribosomes isolated from prokaryotes and eukaryotes into human dermal fibroblasts (HDFs) induces cell cluster formation and transdifferentiation into three germ-layer cells, which is similar to the phenomenon of iPS cell induction through Yamanaka’s factors [29,30,31]. The 80S ribosome is assembled from the 40S and 60S subunits, both of which are composed of ribosomal RNAs (rRNAs) and ribosomal proteins (RPs) [32]. The RPs participate in numerous biological phenomena, including not only protein synthesis, but also tumorigenesis, immune signaling, and development, as extra-ribosomal functions [33,34]. The relationships between the deregulation of RPs and cancers, including colon, prostate, breast, liver, gastric, lung, and brain cancers, have been reported [35,36]. 

From the point of the extra-ribosomal function and cancer, we hypothesized that RPs are molecules that may potentially induce stem-cell-like characteristics in GBM cells. Ribosomal protein S6 (RPS6) has been reported to be associated with cancers, such as leukemia [37], pancreatic cancer [38], and non-small cell lung cancer [39]. Therefore, we focused on RPS6 to investigate the therapeutic resistance of GBM via the induction of stem-cell-like characteristics in GBM cells [36]. In this line of studies, we found that the expression of RPS6 is correlated with the grade of glioma. In RPS6-knockdown experiments, stem-cell-like phenotypes were downregulated; in contrast, these phenotypes were upregulated in the RPS6 overexpression experiments. RPS6 is overexpressed in GSC niches [36]. Moreover, GBM cells form sphere-like structures by incorporating extrinsic ribosomes, wherein cells transdifferentiate into adipocytes and osteocytes [40]. The acquisition of stem-cell-like features and the transdifferentiation potential are regarded to be a result of GBM cell reprogramming.

Here, we focus on and discuss the reprogramming potential and extra-ribosomal function in GBM cells as potential therapeutic targets from the perspectives of plasticity and transdifferentiation potentials. 

## 2. Microenvironments Induce Heterogeneity and Therapeutic Resistance in GBM

GBM is a lethal tumor commonly developing in the adult brain parenchyma, and GBM cells invade the contralateral hemisphere, even in the early stage of tumor progression [41]. Even with complete resection of the enhanced tumor mass lesion in gadolinium-enhanced T1-weighted (Gd-T1WI) magnetic resonance imaging (MRI) followed by chemo-radiotherapies, GBM generally recurs locally [20]. 

To examine the site of GBM recurrence, we retrospectively analyzed Gd-T1WI MRI. The resection rate in 89 consecutive cases of primary GBM was analyzed using Gd-T1WI obtained within 72 h of resection. Complete resection of the enhanced mass lesion was attained in 43 cases (48%); recurrence was observed in 30 of these cases (70%) on the monthly MRI within the follow-up period of 1.5–4.5 years. Local recurrence in the white matter around the tumor removal cavity was observed in 26 cases (87%), while in the gray matter it was observed in 0 cases; distal recurrence was detected in four cases (13%) [20]. Characteristically, oligodendrocyte precursor cells (OPCs) increase at the tumor border and secrete FGF1 and EGF from OPCs to induce stem-cell-like characteristics in glioma cells [20]. These data suggest that some GBM cells dynamically acquire suitable phenotypes to survive and recur in the unique microenvironment of the white matter around the tumor removal cavity.

Clinically, the reactivity of individual patients with GBM to chemo-radiotherapies is different, and different parts of the tumor in the same patients can have varying degrees of sensitivity. Even if the tumor mass can be reduced for some time with chemo-radiotherapy, resistant GBM cells survive and reconstruct the mass lesion later. Many genetic and epigenetic alterations that cause heterogenic phenotypes in GBM have been identified. Several types of heterogeneity in GBM have been reported: (1) intertumoral heterogeneity among tumors of different patients; (2) intratumoral heterogeneity, i.e., different genetic and epigenetic patterns can be detected depending on the site in the same tumor; (3) spatiotemporal heterogeneity between primary and recurrent tumors, depending on the site and time of recurrence [12,13,14,15,42,43,44]. This phenotypical heterogeneity in GBM depends on the high plastic potential.

**Figure 1:**
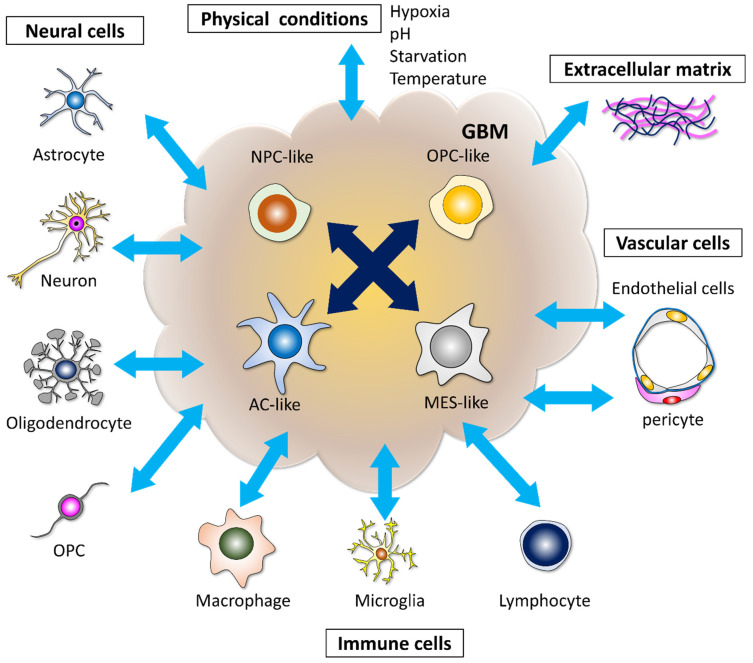
Plasticity and heterogeneity in GBM cells. GBM cells are classified into four subtypes—neural-progenitor-like (NPC-like), oligodendrocyte-progenitor-like (OPC-like), astrocyte-like (AC-like), and mesenchymal-like (MES-like). A single GBM cell can produce all four types of descendants in the xenografted mouse brain under the influence of stimuli from the microenvironment. GBM cells are affected by physical conditions, the extracellular matrix, other GBM cells, and non-GBM cells, including neural cells, immune cells, and vascular cells. The reprogramming of GBM cells is modulated by multidimensional communication in microenvironments.

Recently, data from a single-cell RNA-sequencing analysis showed that human GBM cells can be classified into four subtypes according to the set of gene expression patterns—neural-progenitor-like (NPC-like), oligodendrocyte-progenitor-like (OPC-like), astrocyte-like (AC-like), and mesenchymal-like (MES-like). A single GBM cell can produce all four types of descendants in a xenografted mouse brain [17]. Thus, the dynamic alteration of cell phenotypes in GBM tissue occurs through various kinds of stimuli. GBM cells are affected by the tumor microenvironment constructed with other GBM cells, neural cells, immune cells, vascular cells, extracellular matrix, and physical conditions, and subsequently acquire intercellular heterogeneity (Figure 1). These results explain the limited efficacy of drugs targeting a single pathway in GBM [17]. 

The higher plastic ability of GBM cells leads to intratumoral heterogeneity and therapeutic resistance in GBM. Simultaneously, this higher plastic ability of GBM cells transiting into other subtypes can be termed “reprogramming”. 

## 3. Reprogramming Potential of Glioma Cells

### 3.1. Indirect Reprogramming and Direct Reprogramming of Non-Cancer Cells

**Figure 2:**
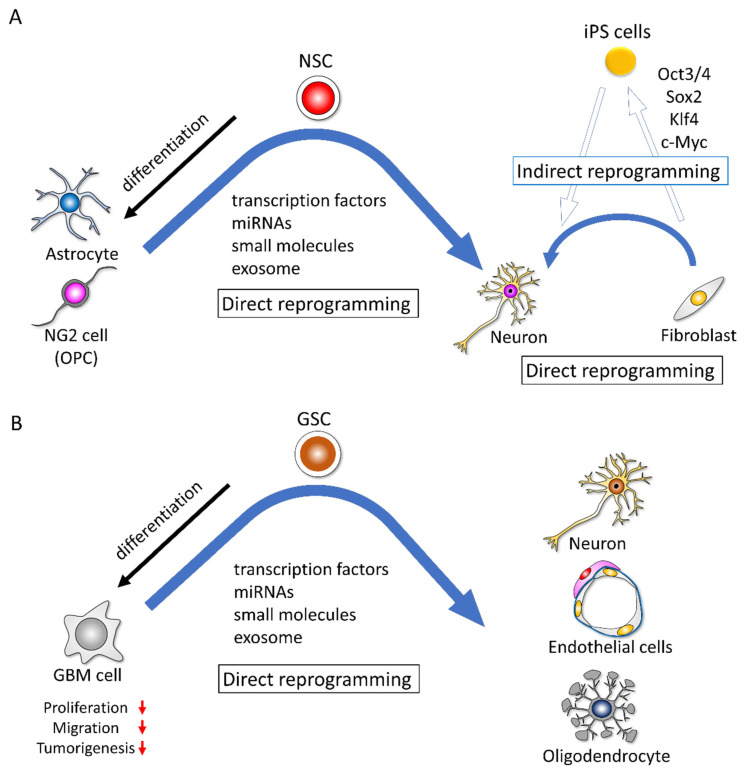
Indirect reprogramming and direct reprogramming in normal cells and GBM cells. (**A**) In indirect reprogramming, pluripotency is induced in fibroblasts by Yamanaka’s factor (Oct3/4, Sox2, Klf4, c-Myc). These iPS cells are then differentiated into three germ layer cells under suitable differentiation culture conditions. In direct reprogramming, fibroblasts, astrocytes, and neuron-glial antigen 2 (NG2) cells (oligodendrocyte progenitor cells (OPCs)) can be directly converted into neurons by transcription factors, microRNAs (miRNAs), small molecules, or exosomes. (**B**) The functions of proliferation, migration, and tumorigenesis are downregulated in the differentiated GBM cells. GBM cells can be directly converted into neurons, oligodendrocytes, and endothelial-like cells.

Takahashi and Yamanaka demonstrated the possibility of developing induced pluripotent stem cells (iPSCs) by reprogramming fibroblasts transfected with four transcription factors—octamer-binding transcription factor 3/4 (*Oct3/4*), sex-determining region Y-box 2 (*Sox2*), Kruppel-like factor 4 (*Klf4*), and *c-Myc*—and then iPSCs can differentiate into all three germ layers cells in chimeric mice (Figure 2A) [30,31]. This discovery has had a major effect on the biology and medical fields. 

Reprogramming can be either “indirect” or “direct” (Figure 2A). Generally, indirect reprogramming means that differentiated somatic cells acquire different cellular phenotypes accompanied by the methylation of DNA and modifications of histones and gene expression profiles [45]. In indirect reprogramming, the cells transit into an iPSC stage and then differentiate into the desired lineage of cells under specific differentiation conditions. In direct reprogramming, the cells convert directly to another cell type with epigenetic or metabolic alterations, bypassing the iPSC stage (Figure 2A) [45,46,47,48]. 

### 3.2. Glioma Cells Possess Potential for Reprogramming and Transdifferentiation

In immunohistochemical analyses, there are cases in which some GBM cells are stained with the neuronal marker MAP2. GBM contains a small population of GSCs that possess the potential to differentiate and express other neural lineage marker genes [5]. The induction of the terminal differentiation of GSCs into neural lineage cells and other lineage cell types is an interesting challenge and should be a potential therapeutic option [49,50,51]. 

A screening study of a kinase inhibitor library revealed that the mechanistic target of rapamycin (mTOR) and Rho-associated coiled-coil-containing protein kinase (ROCK) inhibitors are sufficient to reprogram GBM cells into neurons, which express neuronal markers and generate action potentials and neurotransmitter-receptor-mediated currents [52]. Another study showed that a small-molecule cocktail consisting of forskolin, ISX9, CHIR99021 I-BET 151, and DAPT could turn human GBM cells into terminally differentiated neurons over 13 days. The reprogrammed cells displayed morphological and immunocytochemical characteristics associated with neuronal phenotypes. This chemical cocktail upregulates the expression of neuronal marker genes [53]. Similarly, Gao et al. reported that by using three small molecules (Fasudil (F), a Rho kinase inhibitor; Tranilast (T), a transforming growth factor-β (TGF-β) inhibitor; and TMZ (T)), they (FTT) reprogrammed patient-derived GBM cells to acquire neuronal phenotypes (Figure 2B) [54]. 

Zinc finger protein 117 (ZNF117) was identified from a combination of image-based genome-wide RNAi screening and single-cell RNA-sequencing as a regulator of GSC differentiation. The downregulation of ZNF117 promotes the differentiation of GSCs toward the oligodendrocyte lineage, and the decreased tumorigenic potential influences the JAG2 signaling and regulated NOTCH signaling (Figure 2B) [51].

Unexpectedly, several studies have reported that GSCs can differentiate into endothelial-like cells [55,56,57]. In these studies, the orthotopic injection of patient-derived GSCs into mice produced tumor xenografts with a vasculature composed of human endothelial-like cells. The tumor-derived endothelial-like cells originated from tumor-initiating cells and did not result from cell fusion. Generally, the hypoxia-inducible factor–vascular endothelial growth factor (HIF–VEGF) pathway is important for tumor cell survival and angiogenesis under hypoxic conditions. However, the differentiation of GBM cells into endothelial-like cells is independent of VEGF (Figure 2B) [56]. 

To adapt to hypoxia, GBM cells communicate with their surrounding microenvironment through secreted molecules and vesicles. Under hypoxic conditions, exosomes secreted by GBM cells control the hypoxia-dependent intercellular signaling, where abundant hypoxia-regulated messenger RNAs (mRNAs) and proteins exist [58]. Endothelial cells are reprogrammed by GBM cell-derived hypoxia exosomes in order to secrete several potent growth factors and cytokines and to stimulate pericyte PI3K/AKT signaling, activation, and migration (Figure 2B) [58]. Lucero et al. reported that GBM-derived microRNA (miRNA)-containing EVs induce angiogenesis by reprogramming brain endothelial cells to resemble tumor endothelial cells [59]. These results suggest that GBM cells possess the ability for reprogramming and transdifferentiation into other lineage cells, and exosomes from GBM cells under hypoxic conditions make normal endothelial cells reprogram into GBM tumor endothelial cells. Therefore, GBM cells alter their microenvironment spontaneously and dynamically to survive and multiply.

### 3.3. GBM Cells Acquire an Aggressive Phenotype by Reprogramming through Intercellular Communication

GBM cells acquire an aggressive phenotype to multiply and survive by reprogramming themselves. Genetic and epigenetic alterations are important events for GBM cells. Moreover, GBM cells communicate with other GBM cells and the tumor microenvironment constantly and dynamically. 

Under physiological stress conditions, such as hypoxia and starvation, non-GSC GBM cells acquire GSC-like characteristics and survive through metabolic reprogramming from aerobic to anaerobic glycolysis [60]. Hypoxic conditions promote stem cell marker expression and induce a quiescence state and GSC-like phenotypes related to chemo-radioresistance [61,62,63]. A comparison of RNA expression levels in U87MG cells cultured under hypoxia and those under normoxia showed that genes in the ribosome biogenesis pathway and TNF signaling pathway were enriched [64]. Irradiation itself induces dedifferentiation and increased tumorigenicity in GBM cells [65]. 

**Figure 3:**
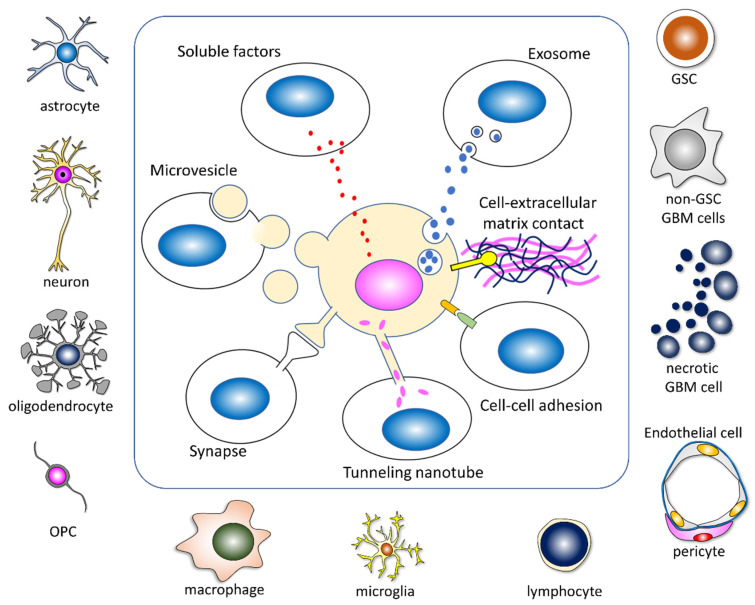
Multidimensional intercellular communication. GBM cells are affected by tumor microenvironments through direct contact and several interventions from GBM cells and non-GBM cells. Some extracellular vesicles contain informative molecules containing proteins, RNAs, DNAs, and surface receptors.

GBM cells communicate with neighboring and distant cells through direct contact and several types of intervention, including some molecules and EVs in the extracellular space (Figure 3). 

Direct cell–cell adhesion, cell–extracellular contact, tumor microtubes, and synapses between GBM cells or non-GBM cells play important roles in reprogramming (Figure 3). The formation of electrochemical networks through synapse-like structures with neurons [26,66], astrocytes [67,68,69], and OPCs [70,71,72] enhances tumor growth. Furthermore, their communication through tumor microtubes confers GBM cells with the advantages of invasion, proliferation, and resistance to radiotherapy [73,74]. The enrichment of synaptic gene expression is mainly found in OPC-like GBM cells [26]. An analysis of the shape of the GBM extension on Gd-T1WI MRI revealed that GBMs extend along the neuronal fibers, such as association and commissural fibers, rather than the capillary network [75]. As oligodendrocytes support neuronal activity and proliferated OPC secret growth factors and cytokines at the tumor border, communication with neurons and oligodendrocytes appears to promote an aggressive phenotype in GBM cells [20,26,66,75]. Astrocytes actively influence the proliferation, migration, invasion, anti-apoptotic ability, chemoprotection, and immunoprotection of GBM cells [76]. Tumor-associated reactive astrocytes interact with GBM cells through ion channels and ion transporters and facilitate tumor progression, aggression, and survival by releasing various cytokines [77].

Several kinds of soluble factors such as growth factors and cytokines are secreted from GBM cells and non-GBM cells via exocytosis. Epidermal growth factor (EGF) and fibroblast growth factor (FGF) are reported as key molecules for neural stem cell and GSC cultures and expand stem cell populations [5,78]. The autocrine function of EGF and FGF sustains the self-renewal of GSCs [79]. As for cytokines, CCL2, referred to as monocyte chemoattractant protein-1(MCP-1), is an important cytokine characterized as a glioma-cell-derived monocyte chemotactic factor [80]. The CCL2–CCR2 axis is highly expressed in GBM and related to the immune escape, angiogenesis, and proliferation of GBM cells; conversely, reduced CCL2 levels are associated with GSC growth inhibition [81,82]. CCL2 is secreted from GBM, immune cells, and oligodendrocytes, which interact mutually [83,84]. Regretfully, we omitted many other important growth factors, including cytokines and chemokines [84,85,86,87,88].

EVs including microvesicles, exosomes, apoptotic bodies, and oncosomes transfer some informative molecules containing proteins, RNAs, DNAs, and surface receptors to target cells, and this promotes the reprogramming of GBM cells and therapeutic resistance [19,20,21,89,90,91,92,93]. GBM-derived EVs induce the tumor-promoting phenotype in NSCs [94]. Several key genes, including *S100B*, *CXCL14*, *EFEMP1*, *SCRG1*, *GLIPR1*, and *CD44*, and dysregulated signaling are linked to the transformation of NSCs [94]. Exosomes derived from GSCs can reprogram non-GSC into GSCs [95]. The ability for proliferation, sphere-formation, invasive capacities, and tumorigenicity in non-GSC GBM cells is upregulated substantially after GSC exosome treatment [95]. Gao et al. reported that the transfer of genetic material is achieved mainly through EVs, although cell fusion also plays a minor role, and individual GBM cells communicate with distinct sets of non-GBM cells [96].

Moreover, EVs released by GBM cells stimulate normal astrocytes to acquire a tumor-supportive phenotype that promotes migration and invasion, enhances cytokine production, and activates tumor cell growth [97,98]. Moreover, in a soft agar assay, EVs derived from GBM cells showed a transformative effect on normal human astrocytes [98]. GBM-derived EVs promote the neoplastic growth of pre-transformed astrocytes but not normal human or mouse astrocytes through metabolic reprogramming. GBM EV-mediated reprogramming is partially associated with the transfer of full-length mRNAs encoding RPs, oxidative phosphorylation, and glycolytic factors [99]. Thus, through reprogramming, various types of stimuli promote aggressive phenotypes in GBM cells and modulate tumor-supportive phenotypes in non-GBM cells to reconstruct beneficial microenvironments as GSC niches.

## 4. Deregulation of Ribosome Biogenesis in Cancer

**Figure 4:**
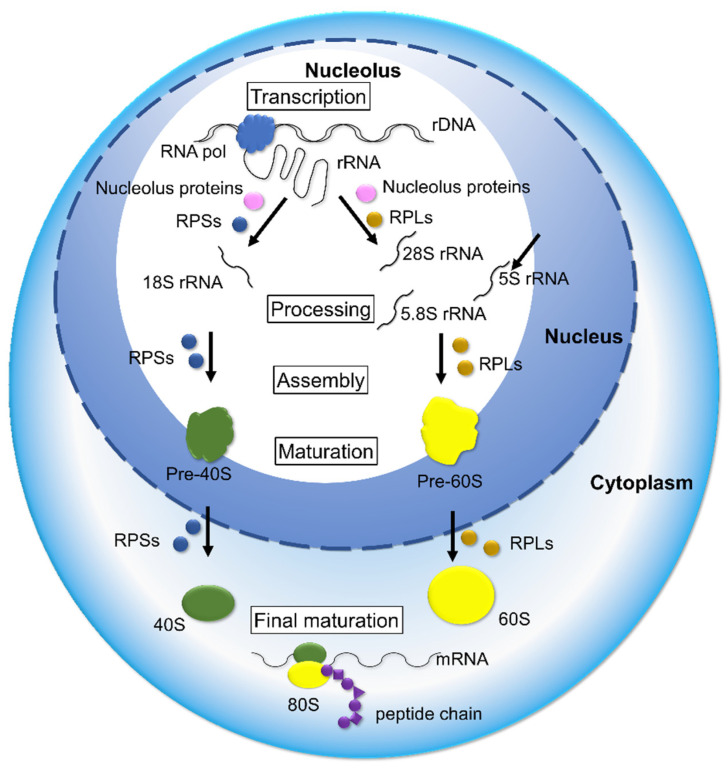
In ribosome biogenesis, many molecules participate in multiple steps, including rDNA transcription, rRNA processing, nucleolus protein assembly, ribosomal proteins, rRNAs, the maturation of large and small ribosomal subunits, and finally the maturation of 80S ribosome. The deregulation of ribosome biogenesis causes an abundance or shortage of ribosomal proteins, which induce tumor-promotive or -suppressive effects in cancer.

Human ribosomal biogenesis includes several steps, such as the transcription of ribosomal DNA (rDNA), processing and modification of ribosomal RNA (rRNA), and assembly and maturation of the 40S and 60S subunits. The small 40S subunit is constructed with 18S rRNA and 33 RPs, and the large 60S subunit is formed with three rRNAs (28S, 5.8S, and 5S) and 48 RPs, then finally small and large subunits are assembled. Consequently, mature 80S ribosomes are constructed, which function in protein synthesis (Figure 4) [100,101,102]. Therefore, RPs are essential molecules for constructing ribosomal subunits and mature ribosomes. In ribosome biogenesis, TP53, PTEN, and Rb function as potent ribosome biogenesis suppressors [103,104]; conversely, PI3K-Akt-mTOR, c-MYC, RAS-MAPK, and NF-κB function as activators [105,106,107,108,109].

Interestingly, the deregulation of ribosome biogenesis exhibits a paradoxical function from a hypo-proliferative cellular response to a hyper-proliferative oncogenic phenotype, which was referred to as Dameshek’s riddle [110]. Ribosome insufficiency leads to ribosome misassembly, the dysregulation of protein synthesis, and oncogenic protein expression; moreover, some RPs regulate major cancer proteins [101,111]. 

**Figure 5:**
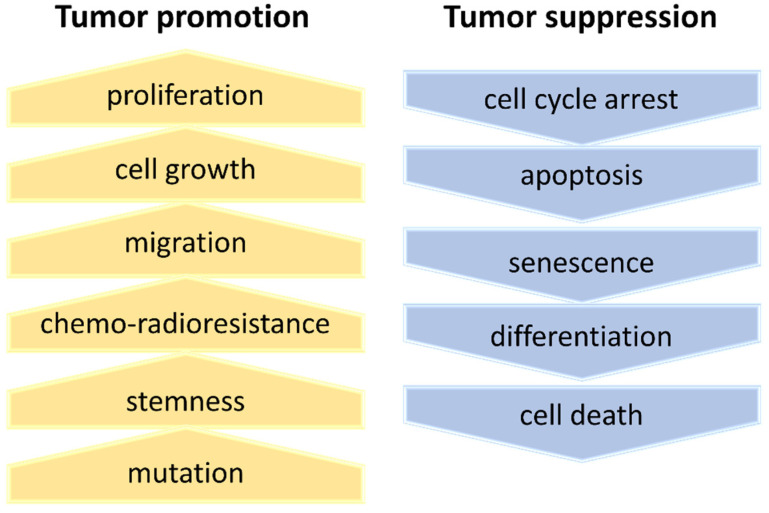
Extra-ribosomal function in cancer. The deregulation of ribosome biogenesis induces tumor-promotive and -suppressive functions.

Ribosome-free RPs are known to have several functions. RPs are related to a variety of aspects of carcinogenesis; the positive effects in cancer progression are the upregulation of the proliferation and migration potential and the induction of stemness. Conversely, the negative effects are induced cell cycle arrest, apoptosis, and cellular senescence as extra-ribosomal functions [33,34,35,36,40,101,112,113,114,115] (Figure 5). 

The tumor-suppressive effects of extra-ribosomal functions involve nucleolar stress, which activates the RP-MDM2-p53 pathway with the consequent sustained p53 stabilization, which induces cell cycle arrest, apoptosis, and cell death [33,114,116,117]. Impaired ribosome biogenesis through the deregulation of polymerase I transcription, rRNA processing, ribosome assembly, and transport promote tumor-suppressive functions, including cell cycle arrest, apoptosis, senescence, dormancy, differentiation, and cell death (Figure 5). 

In addition, the p53-independent pathway has been reported, and it is involved in c-MYC inhibition by RPL5, RPL11, and RPS14, while other tumor-suppressive functions are regulated by nucleolar proteins such as nucleophosmin (NPM) and ARF [101,118,119,120,121]. 

In contrast, the tumor-promotive functions upregulate cell proliferation, growth, migration, chemo-radioresistance, and stemness (Figure 5). Moreover, the nucleolar stress conditions through the deregulation of ribosome biogenesis also promote metabolic reprogramming and secondary mutation [111]. 

Ribosomopathies are rare inherited diseases, wherein genetic mutations in ribosome biogenesis reduce the ribosome levels [122]. Patients with ribosomopathies have a higher risk of developing cancer [111]. Ribosomopathies are divided into two major classes based on their predispositions to cancer [122,123,124]. Patients with inherited bone marrow failure, including Diamond–Blackfan anemia, Shwachman–Diamond syndrome, and dyskeratosis congenita, show a high predisposition to cancer, but those with Treacher–Collins syndrome do not [122,123,124]. 

## 5. Ribosome Incorporation Induces Reprogramming in Somatic Cells

Tumor-suppressive and -promotive functions of RPs in cancer have been discussed as extra-ribosomal functions. Additionally, Ito et al. reported that incorporating ribosomes into adult HDFs promotes reprogramming and multipotency, which is a novel and interesting finding regarding ribosome function [29]. Originally, Ohta had reported that adult HDF incorporation of lactic acid bacteria (LAB) resulted in the formation of embryoid-body-like cell clusters similar to embryoid bodies derived from ES cells and expressed a set of pluripotent markers, including Nanog, Sox2, Oct3/4 and Tdgf1. These cells differentiated into all three germ layer cells [125]. An investigation of factors in LAB that induce multipotency showed that ribosome incorporation into HDFs promotes dedifferentiation and transdifferentiation potentials [29]. Through ribosome incorporation, HDFs form ribosome-induced cell clusters (RICs), wherein cells express pluripotency marker genes. Moreover, dedifferentiated cells in RICs can differentiate into the three germ-layer cells, such as ectodermal neurons, mesodermal cardiomyocytes, endodermal hepatocytes, adipocytes, and chondrocytes [29]. Ribosomes from various prokaryotes (Gram-positive and -negative bacteria) and eukaryotes (yeast, mouse, and human) promote RIC formation [29]. These ribosome-mediated reprogramming potentials were not related to the translational activity of the incorporated extrinsic ribosomes [29,126]. Based on these interesting results, we hypothesized that incorporating ribosomes promotes plasticity and reprogramming in GBM cells, through which GBM cells acquire GSC-like properties and therapeutic resistance.

## 6. Incorporation of Ribosomal Proteins S6 Induces Reprogramming in Glioma Cells

To clarify whether the incorporation of ribosomes promotes stem cell properties and therapeutic resistance in glioma cells, investigations focusing on RPS6 were performed because the overexpression and phosphorylation of RPS6 have been reported in various types of cancers, including acute myeloid leukemia [37,127], non-Hodgkin lymphoma [128], oral squamous cell carcinoma [129], non-small cell lung cancer [39], breast cancer [130], gastric cancer [131], pancreatic cancer [38], renal cell carcinoma [132], ovarian cancer [133], melanoma [134], and others [112]. RPS6 processes 30S pre-rRNA into 18S rRNA, which is used to form the small 40S subunit. Thereafter, the small 40S and large 60S subunits assemble and form mature 80S ribosomes [135]. 

**Figure 6:**
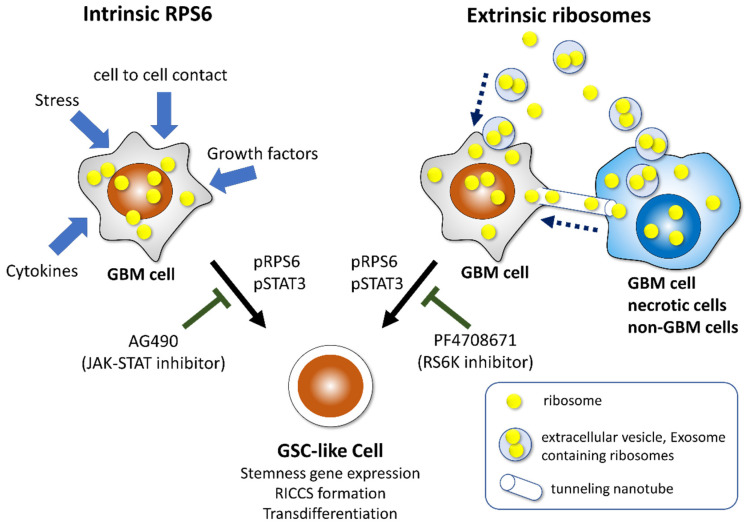
Intrinsic RPS6 and extrinsic ribosomes induce GSC-like phenotypes in GBM cells. Multiple stimuli in the tumor microenvironment induce ribosome biogenesis. The overexpression of RPS6 increases stemness gene expression and RICCS formation, which is suppressed by AG490 (JAK-STAT inhibitor). GBM cells communicate with other GBM cells, necrotic cells, and non-GBM cells through extracellular vesicles, including exosomes and tumor nanotubes. Ribosome incorporation promotes stemness gene expression and RICCS formation, which is suppressed by PF4708671 (RPS6K inhibitor). Cells in RICCS acquire transdifferentiation potentials.

The immunohistochemical analysis showed significant upregulation of RPS6 expression in high-grade glioma compared with that in low-grade gliomas [36]. The sphere-forming ability and expression of the stem cell marker genes *Nestin* and *Sox2* were downregulated by the knockdown of RPS6, whereas the sphere-forming ability increased in overexpression experiments in U251MG and U87MG glioma cell lines [36], both of which were classified as GBM, IDH-wildtype. RPS6-specific siRNA reduced the sphere-forming ability and expression of *Nestin* and *Sox2* and phosphorylated STAT3 (pSTAT3). The Janus kinase/signal transducers and activators of transcription (JAK/STAT) inhibitor (AG490) suppress the sphere-forming ability. Moreover, an immunohistochemical analysis showed that the RPS6 expression was predominant in the perivascular, perinecrotic, and border niches of GBM tissues [36]. To confirm these data, we used the Ivy Glioblastoma Atlas Project database, which provided gene expression data via the microdissection of several areas regarded as GSC-dominant [136]. The expression of RPS6 is significantly higher in GSC-dominant areas, such as the site of microvascular proliferation, pseudopalisading cells around necrosis, infiltrating tumors, and the leading edge [36]. RPS6 is predominantly present in sites containing GSC niches. Similar to RPS6 expression, many other ribosomal proteins show higher expression in the GSC-dominant sites. Thus, the intrinsic RPS6 can induce stem-cell-like properties in glioma cells (Figure 6) [36].

Moreover, to investigate the effects of extrinsic ribosomes on phenotype alterations in glioma cells, we harvested ribosomes derived from prokaryotes and U251MG using ultracentrifugation. Ribosomes derived from prokaryotes were added to the culture dish where U251MG cells were plated in DMEM/F12 (serum-free and growth-factor-free). These GBM cells formed ribosome-induced cancer cell spheroids (RICCS) and showed increased expression of the stemness genes *Nestin* and *Sox2*. In the differentiation analysis, these cells in RICCS could be transdifferentiated into adipocytes and chondrocytes via culturing in a differentiation-specific medium of each cell type [40]. These effects were modulated through intrinsic RPS6 phosphorylation. These effects were interrupted by an inhibitor of RPS6 signaling (PF4708671, ribosomal S6 kinase inhibitor). The extrinsic ribosomes derived from GBM cells induce the formation of RICCS and the expression of RPS6, phosphorylated RPS6, and the stemness genes *Nestin* and *Sox2*. These effects of the ribosome incorporation were interrupted by an inhibitor of RPS6 signaling (PF4708671, ribosomal S6 kinase inhibitor) [40]. These results demonstrated that intrinsic and extrinsic RPS6 can promote the induction of stem-cell-like characteristics in GBM cells [113]. This ability for stemness induction is a novel extra-ribosomal function of RPS6 (Figure 6) [36,40,113].

Taken together, GBM cells that are cultured for long periods with serum could be reprogrammed and could acquire transdifferentiation potential via ribosome incorporation, resembling the phenotypes of RICs derived from HDFs. Thus, the reprogramming ability caused by ribosome incorporation appears to be conserved in normal cells and cancer cells.

## 7. Deregulation of Ribosome Biogenesis Modulates Aggressiveness in GBM

The expression levels of several ribosomal proteins in GBM have been reported (Table 1). RPS27, also called metallopanstimulin-1, is a component of the 40S subunit of ribosomes, which is highly expressed in the various types of tissues, including malignancies in the colon [137], prostate [138], breast [139], and stomach [140]. An increased RPS27 level in the serum has been identified; this is a useful marker for the early detection of various types of cancer [141]. As for brain tumors, in a previous study, the mRNA of RPS27 was 6.2- and 8.8-fold (mean) enhanced in gliomas of WHO grades II and III with (*p* < 0.01) and without IDH mutation (*p* = 0.01), respectively, compared with that in the normal healthy brain. Additionally, GBM displayed a 4.6-fold increased mean expression (*p* = 0.02). However, the expression of RPS27 was not related to the WHO grade in gliomas [142]. In the findings of an analysis using the IVY GAP database, the expression of RPS27 was dominantly detected in the area of microvascular proliferation and in pseudopalisading cells around necrosis, but not in the infiltrating area of tumor cells. The levels of RPS27 were not related to the progression-free survival or the overall survival of patients with GBM [142]. Moreover, how RPS27 functions in gliomagenesis remains unclear.

RPS15A is overexpressed in GBM tissues. The knockdown of RPS15A inhibits cell proliferation and colony formation and induces apoptosis in U251 cells [143]. Correspondingly, the knockdown of RPS15A suppressed tumorigenesis in xenograft models via the Akt pathway [144].

RPL34 is a component of the pre-ribosome 60S subunit [145]. The expression of RPL34 is significantly higher in GBM than in low-grade gliomas and the normal brain and is related to poor survival and the proliferation of GBM cells [145].

Conversely, some ribosomal proteins play a role in suppressing tumorigenesis by activating some tumor suppressors and inactivating oncoproteins [33]. Heterozygous deletion or mutation of RPL5 was found in 11% of GBM [146]. Clinically, patients expressing low levels of RPL5 have been shown to have shorter 5-year overall survival and mean survival times (13.8 months, *n* = 414) than those expressing high levels of RPL5 (14.7 months, *n* = 442) [146]. Thus, RPL5 has a tumor-suppressive function in GBM.

RPS11, RPS16, and RPS18 influence the susceptibility of GBM cells to topoisomerase II inhibitor (etoposide) treatment, and a loss of RPS11 leads to resistance to etoposide [147]. Under cellular stress conditions, intrinsic ribosome biogenesis is increased, and ribosome-free RPs promote GSC-like phenotypes and aggressiveness in GBM cells as extra-ribosomal functions. 

Meanwhile, the mechanisms of incorporating extrinsic RPs into GBM cells have not yet been revealed. Thus, some questions remain regarding how RPs are secreted and incorporated into GBM cells in the tumor microenvironment. 

In RPS6, approximately 5% of endogenous RPS6 is detected in ribosome-free subcellular fractions [148]. Regarding the secretion of RPs, Kim et al. reported that protein secretion is a general phenomenon by which cells communicate with the extracellular environment, and RPS3 is secreted in several cancer cell lines such as HT1080 (human fibrosarcoma) and MPC11 (mouse plasmacytoma). The secreted RPS3 level increased in doxorubicin-resistant MPC11 cells compared with that in original MPC11 cells [149]. 

EVs transfer some informative molecules containing proteins, RNAs, DNAs, and surface receptors to target cells. GBM EVs contain full-length mRNAs encoding RPs, are involved in oxidative phosphorylation, and act as glycolytic factors [99]. Exosomes containing RPs from Schwann cells are transferred to axons [150,151]. EVs, including exosomes, appear to be important vehicles for the intercellular communication of ribosomes. Tunneling nanotubes, which are thin, membranous, open-ended tubes, are another candidate because they directly transfer cellular materials, including mitochondria, to GBM cells [152].

## 8. Incorporation of Ribosome Induces Reprogramming and Transdifferentiation Potential in Cancer Cells

The overexpression of intrinsic RPS6 induces GSC-like phenotypes. Moreover, extrinsic ribosome incorporation promotes GSC-like phenotypes and the reprogramming and transdifferentiation potentials in GBM cells linked to pRPS6 and pSTAT3 [36,40]. Similarly, extrinsic ribosome incorporation induces reprogramming and transdifferentiation potentials in several types of cancers. 

When extrinsic ribosomes purified from *Escherichia coli* strain JE28 were transferred into human breast cancer cell line MCF7, the cell proliferation potential decreased and the population in the G0 phase increased, while cyclinD1 expression disappeared in RICCS on day 20 in culture, but those in control cells remained. These data suggest that cell cycle arrest was induced by ribosome incorporation. During RICCS formation, the expression of *TGF-*β and *Snail*, which is a marker gene in epithelial–mesenchymal transition, was upregulated from 1 h to day 1 and then downregulated, although the expression of E-cadherin was similar to that in the control, resulting in incomplete EMT inhibition. The autophagy pathway is activated by ribosome incorporation. These results demonstrated that ribosomal incorporation induces cell cycle arrest and reprogramming, which alters the phenotypes of MCF7 [153].

Through extrinsic ribosome incorporation, the non-small cell lung cancer cell line, A549, and gastric tubular adenocarcinoma cell line, H-111-TC, also formed RICCS within 2–3 days, and both cells showed transdifferentiation potentials into adipocytes and osteoblasts in the specific induction medium. Ribosome incorporation in the A549 cell line gradually upregulated cell proliferation marker Ki67, but the expression of *EGFR* and *cyclicD1* peaked on day 7 and then decreased on day 14; however, that of *CXCR4* increased until day 14 [154]. 

Ribosomes derived from eukaryotic, prokaryotic, and GBM cells promote RICCS formation in glioma cells, breast cancer cells, lung cancer cells, and gastric cancer cells, and these RICCS cells acquire transdifferentiation potentials [40,153,154]. During the senescence-like state, RICCS are reprogramed and the state is reversed under the stimuli of the differentiation induction medium, resulting in temporal proliferation and transdifferentiation [126]. Therefore, reprogramming by ribosome incorporation is conserved in several types of cancer (Table 2). 

## 9. Ribosome Biogenesis as the Therapeutic Target for GBM

Generally, increased ribosome biogenesis and protein synthesis are observed during the growth of normal tissues as a result of the expansion of the size and number of cells. However, the deregulation of ribosome biogenesis in cancer is observed, increasing oncogenic and decreasing tumor-suppressive ribosomes. 

The functions of ribosomal proteins are still intricate; however, the potential to induce aggressive phenotypes and transdifferentiation ability by reprogramming in GBM cells is considered a novel therapeutic target.

Investigations focusing on ribosome biogenesis as the therapeutic target in GBM have been conducted. Ribosome-inactivating proteins (RIPs) form a large family and are found in several plants, which inactivate the 60S ribosomal subunits and are used in chemotherapeutic agents for cancers [155]. Combination therapy with quinoin, a type 1 RIP from quinoa seeds, and temozolomide has shown synergistic cytotoxic effects in GBM cells [156].

Pescadillo ribosomal biogenesis factor 1 (Pes1), block of proliferation 1 (BOP1), and WD repeat domain 12 (WDR12) play crucial roles in the ribosome biogenesis pathway. The Pes1-BOP1-WDR12 complex modulates pre-rRNA processing for the maturation of 28S and 5.8S rRNAs [157,158,159]. Pes1 is related to tumor cell proliferation, invasion, and metastasis in various types of cancers [159]. WDR12 is an essential factor for processing the 32S pre-rRNA. WDR12 is required for ribosome biogenesis in GSCs, and higher expression of WDR12 has been observed in GSCs than in non-GSCs and normal brain cells [160]. The increased expression of WDR12 is related to the progression of GBM and shorter overall survival [160]. Silencing WDR12 via small hairpin RNA induces the degradation of the PeBoW complex and suppresses the maturation of 28S rRNA [158]. A lack of WDR12 inhibits GSC proliferation and tumor growth and prolongs the survival time of mice-injected GSCs [160,161]. 

Similarly, the inhibition of the purine guanosine monophosphate biosynthesis decreases the production of rRNA and GBM cell growth [162]. Purine metabolism is an important key mediator of DNA repair and radiation resistance in GBM [163]. GBM cells depend on the de novo biosynthesis of pyrimidines to support the rDNA transcription and cell growth. However, the effects of the inhibition of pyrimidine biosynthesis in normal cells are not sufficient for pyrimidine synthesis, rRNA production, and proliferation [164]. To generate rRNA, GBM cells strongly depend on the de novo pyrimidine biosynthesis pathway; therefore, the specific inhibitor of this pathway is an expected therapeutic target of GBM [164]. The effects of brequinar (dihydroorotate dehydrogenase (DHODH) inhibitor) are specific to GBM cells, and it may be a safe drug with minimal adverse effects [164].

ErbB3 expression correlates with an increased expression of the 47S ribosome precursor and cell proliferation [165]. ErbB3/C23 (nucleolin) interferes with RNA polymerase I activity and 47S rRNA synthesis. Actinomycin D binds to ErbB3/C23 (nucleolin) and induces nuclear accumulation of ErbB3/C23, which inhibits rRNA transcription [165]. Actinomycin D induces nucleolar stress and is a new treatment strategy for GBM [165].

STAT6 is downregulated in human GBM specimens. Under hypoxic conditions, the expression of STAT6 is downregulated, and HIF-1αprotein synthesis is increased through the activation of the mTOR/S6K (RPS6 kinase)/S6 (RPS6) signaling pathway, which promotes cell survival and maintains GSC phenotypes [166]. The expression of p-mTOR and p-RPS6 is substantially suppressed by the Jinlong capsule, and the inhibition of mTOR reduces cell migration and the invasion ability in GBM cells [167].

Alpha Thalassemia/X-linked mental retardation syndrome (ATRX) mutation is one of the important genes for diagnosing gliomas. ATRX maintains rDNA heterochromatin formation and stability, and ATRX loss of function contributes to tumorigenesis through the rDNA instability [168].

Many chemotherapeutic drugs target the process of ribosome biogenesis, and each drug inhibits ribosomal RNA synthesis at the stage of (i) rRNA transcription (e.g., oxaliplatin, doxorubicin, mitoxantrone, methotrexate), (ii) early rRNA processing (e.g., camptothecin, flavopiridol, roscovitine), or (iii) late rRNA processing (e.g., 5-fluorouracil, MG-132, homoharringtonine) [169]. 

Several compounds, including CX-3543 [170], CX-5461 [171], and BMH-21 [172], inhibiting pol I have been developed. A clinical trial for CX-5461 showed that p53 wild-type leukemia cells are more sensitive, while p53 mutant cells can also be responsive [173,174,175].

## 10. Conclusions and Future Directions

The elucidation of the mechanisms underlying the acquisition of stemness in GBM cells is a fundamental clinical issue for improving the prognosis of patients with GBM. In this review, we described the possibility of increasing intrinsic ribosomes by deregulating ribosome biogenesis and incorporating extrinsic ribosomes that promote reprogramming in GBM cells, which induces aggressiveness, chemo-radioresistance, and transdifferentiation potential in GBM cells. However, we could realize and develop new therapeutic approaches. Regulating ribosome biogenesis in GBM cells induces (i) nucleolar stress and hypo-proliferative tumor-suppressive phenotypes and (ii) induces a terminally differentiated phenotype and converts malignancy to benignity with epigenetic modifications [45]. Indeed, some chemotherapeutic drugs targeting ribosome biogenesis have been studied [101,143,176]. Notably, reprogramming GBM cells differentiated into neurons using ROCK-mTOR inhibitors or three small molecules (FTT) significantly suppressed the tumor growth and prolonged the survival time in xenografted mice [52,54]. The downregulation of ZNF117 promoted the differentiation of GSC toward the oligodendrocyte lineage and decreased the tumorigenic potential [51]. There is a possibility that small molecules regulate the GSC differentiation into desired lineage cells in the future.

The dynamic phenotype alteration of GBM cells complicates chemoradiotherapies, which also demonstrates a possibility that different subtypes of GBM cells acquire distinct phenotypes through the reprogramming process. There are several hurdles that need to be overcome before clinical trials of cancer cell reprogramming therapy, such as clarifying the mechanism, efficacy, safety, and delivery methods for such agents. However, the transdifferentiation of GBM cells into other neural lineage cells or benign cells or hypo-proliferative states, including senescence, apoptosis, and cell death, may be a feasible challenge for future studies. 

**Figure 7:**
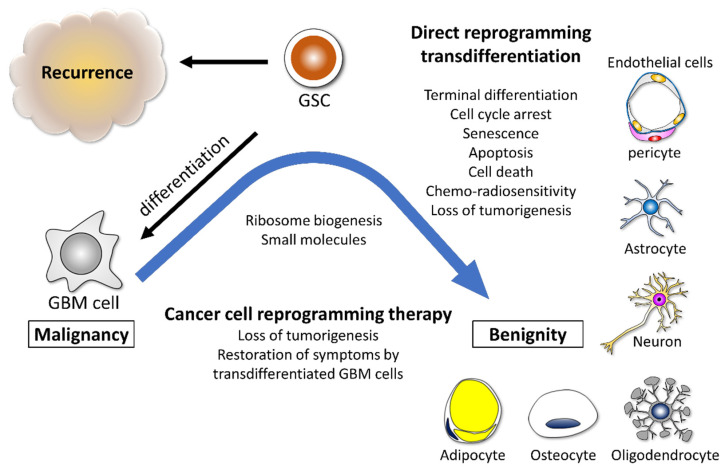
Cancer cell reprogramming therapy might convert malignancy to benignity. In reprogramming possesses, malignant phenotypes convert to benign phenotypes. By inducing terminal differentiation, a hypo-proliferation state, chemo-radiosensitivity, and tumorigenicity loss, GBM cells are eliminated or they exist as non-proliferating cells in the brain. Ideally, if terminally differentiated GBM cells possess normal neuronal functions and are integrated into a normal brain, tumor deletion and regenerative medicine to improve neurological deficits could coexist.

Therefore, through further knowledge on reprogramming in GBM cells, the residual GBM cells might be differentiated into non-proliferating functional cells. Moreover, if neurons derived from GBM cells can reconstruct neural networks, an improvement of neurological deficits might be expected (Figure 7). The transdifferentiation of GBM cells into non-malignant cells through cancer cell reprogramming therapy might contribute to future GBM treatments.

## Figures and Tables

**Table 1:** Ribosomal protein in glioblastomas.

Ribosomal Protein	Function	Induced Phenotypes	Reference
RPS6	Oncogenic	Sphere-forming ability Stemness gene expression (*Nestin*, *Sox2*) Higher expression in GBM Higher expression at GSC-dominant area	[34,38]
RPS27	Oncogenic	High expression in gliomas Higher expression at GSC-dominant area No relation to survival time	[136]
RPS15A	Oncogenic	Higher expression in GBM Proliferation Colony formation Anti-apoptosis Tumorigenesis Poor survival	[137,138]
RPL34	Oncogenic	Higher expression in GBM Proliferation Anti-apoptosis Poor survival	[139]
RPL5	Tumor-suppressive	Mutation 2.5%, deletion 8.4% in GBM Poor survival time in low RPL5 expression	[140]
RPS11	Tumor-suppressive	High expression levels mean high susceptibility to topoisomerase II inhibitors (etoposide and doxorubicin)	[141]
RPS16			
RPS18			

**Table 2:** Ribosome incorporation into cancer cells.

Cell Line	Ribosome	Alteration of Phenotypes	Reference
Glioblastoma U251MG	Prokaryote	RICCS formation Stemness gene expression (*Nestin*, *Sox2*) pRPS6, RPS6 expression pSTAT3 expression Transdifferentiation (adipocyte, osteocyte)	[38,107]
	Eukaryote (U252MG)	RICCS formation Stemness gene expression (*Nestin*, *Sox2*) pRPS6, RPS6 expression pRPS6 co-expressed Nestin RPS6K inhibitor (PF4708671) suppresses RICCS formation RPS6K inhibitor (PF4708671) suppresses the expression of *Nestin* and *Sox2*	[38,107]
Breast cancer MCF7	Prokaryote	RICCS formation Increased G0 and early G1 phase cells EMT-like phenomenon Autophagy pathway activation p53-mediated stress response	[120,147]
Non-small cell lung cancer A549	Prokaryote	RICCS formation Transdifferentiation (adipocyte, osteoblast) EGFR expression was increased on day 7, then decreased on day 14 CXCR4 expression was increased on day 14 Ki67-positive cells increased gradually on day 14 cyclinD1 expression increased by day 7, then decreased by day 14 In the tumor-forming assay, direct injection of ribosomes into the tumor mass No significant difference in tumor size and volume between control and ribosome-incorporated tumor	[120,148]
Gastric tubular adenocarcinoma H-111-TC	Prokaryote	RICCS formation Transdifferentiation (adipocyte, osteoblast)	[120,148]

## Data Availability

Not applicable.
